# Superficial Spread of Nonkeratinizing Squamous Cell Carcinoma of the Cervix to the Endometrium: A Case Report

**DOI:** 10.7759/cureus.63931

**Published:** 2024-07-05

**Authors:** Varsha Rangankar, Siddharth Bokil, Sanika Deshmukh

**Affiliations:** 1 Radiodiagnosis, Dr. D. Y. Patil Medical College, Hospital & Research Centre, Pune, Pune, IND

**Keywords:** endomertrium, locally advanced cervical cancer, superficial spread, pelvic mri, squamous cells carcinoma

## Abstract

Squamous cell carcinomas (SCCs) are the most prevalent malignant tumors affecting the cervix. The superficial spread of SCC along the inner surface of the uterus, replacing the endometrium with malignant cells, is a rare subtype of cervical cancer. We present the case of a 55-year-old woman who complained of per-vaginal white discharge and generalized weakness for one month. Clinical examination revealed a bulky and fibrosed cervical os. A cervical biopsy confirmed the diagnosis of poorly differentiated nonkeratinizing SCC. MRI showed an endocervical infiltrative, heterogeneously enhancing mass lesion involving the cervix, along with cervical stenosis and hydrometra. Irregular thickening with nodular enhancing deposits showing morphology similar to the cervical lesion and restricted diffusion were noted along the endometrial lining contiguous with the cervical lesion. The patient underwent a radical hysterectomy, and histopathological examination revealed poorly differentiated nonkeratinizing squamous cell cervical carcinoma with contiguous squamous cell extension into the uterine endometrium, confirming the diagnosis of superficially spreading cervical SCC. Establishing the continuity of the lesion on imaging and histopathological testing is critical to confirm the presence of a superficial spread of cervical cancer and rule out contemporaneous endometrial cancer.

## Introduction

Cervical cancer remains a significant health burden globally, with squamous cell carcinomas (SCCs) representing the predominant histological subtype, accounting for 70-80% of cases [1]. While the invasion of the uterine wall by cervical SCC is a common feature, superficial spreading SCC, characterized by its unique spread to the inner surface of the uterus and replacement of the endometrium with cancerous cells, is exceedingly rare [1]. While the literature on superficially spreading SCC is limited, it is imperative to recognize this distinct entity due to its unique clinical presentation and management considerations [2].

This case report describes the clinical course of a 55-year-old woman who was diagnosed with poorly differentiated nonkeratinizing SCC of the cervix with superficial dissemination. The patient initially came with a per-vaginal white discharge and generalized weakness. Through this case report and pertinent findings from the literature, we hope to clarify the clinical features, diagnostic difficulties, and treatment implications of superficially spreading SCC. We aim to enhance the current knowledge base and enable better diagnostic and treatment strategies for individuals with comparable clinical situations by bringing attention to this uncommon but clinically noteworthy entity.

## Case presentation

A 55-year-old female came to our tertiary care hospital with complaints of per-vaginal white discharge associated with generalized weakness of one-month duration. She had attained menopause 14 years ago, before which she had normal, regular cycles of 3-4/30 days. She had two full-term vaginal deliveries, which were uneventful. There was no significant family history or history of any comorbidity or addiction.

On general examination, the patient was afebrile with a pulse rate of 80 beats per minute and a blood pressure of 130/70 mm Hg. Systemic examinations, including the central nervous system and cardiorespiratory system, were within normal limits. The abdomen was soft and nontender on palpation. Routine blood tests revealed normal blood cell count. Serological examinations for hepatitis B, hepatitis C, human immunodeficiency virus (HIV) and human papillomavirus (HPV) were negative. On a per-vaginal speculum examination, the cervix appeared bulky, while the cervical os was found to be fibrosed and stenosed. The posterior fornix was not freely mobile, but the bilateral lateral fornices were free. The patient underwent a cervical biopsy, and histopathological examination revealed poorly differentiated, nonkeratinizing SCC of the cervix. Clinically, she was classified as having FIGO stage II B cervical cancer.

**Figure 1:**
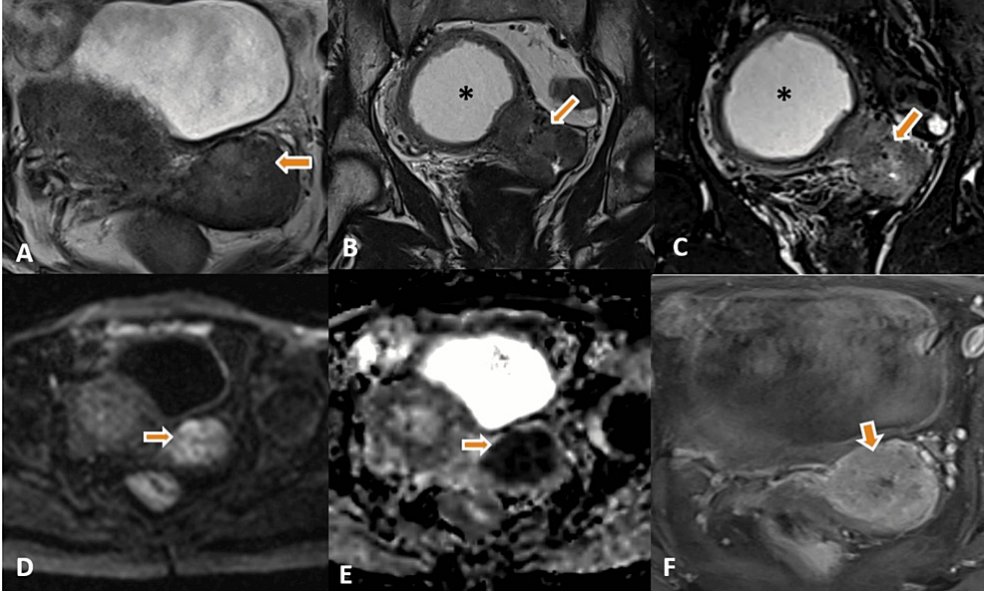
A-F: Axial (A) and coronal (B) T2-weighted and STIR coronal (C) images show endocervical infiltrative heterogeneously high signal intensity lesion involving cervix (thick arrows). Significant cervical stenosis with resultant hydrometra and an enlarged uterus is also seen (B, C asterisk). The lesion shows significant diffusion restriction (D, thick arrow) and a correspondingly low signal on ADC images (E, thick arrow). The axial post-contrast fat-saturated T1-weighted image shows heterogeneous enhancement of the lesion in the cervix (F, thick arrow).

**Figure 2:**
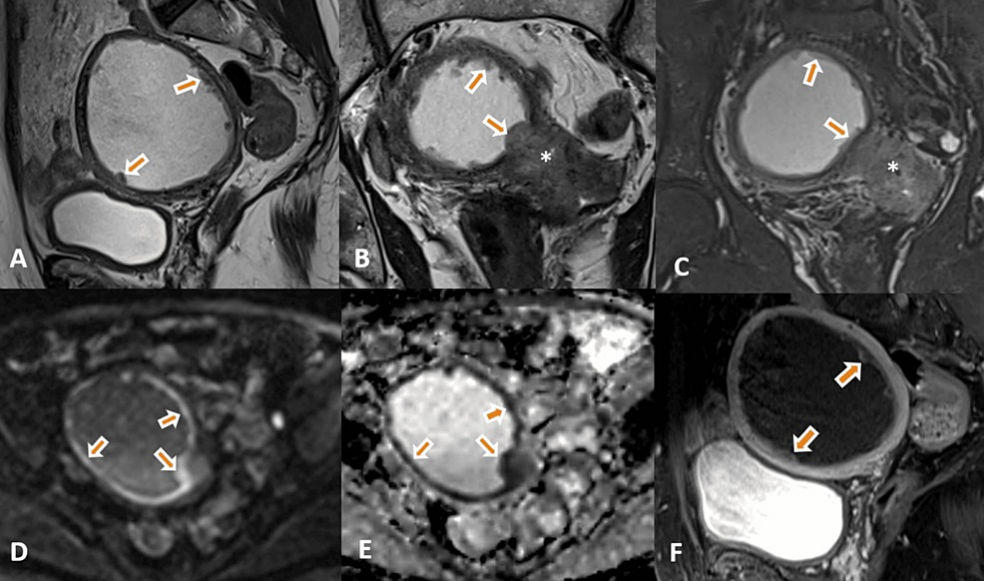
A-F: T2-weighted sagittal (A) and coronal (B) and STIR sagittal (C) images show irregular thickening and nodular deposits along the endometrial lining (thick arrows), in contiguity with the cervical lesion, and signal intensity to the primary cervical lesion (B, C asterisk). The thickened endometrium and nodular deposits show diffusion restriction (D, thick arrows) and corresponding low signal on the ADC image (E, thick arrows) with heterogenous enhancement on the axial post-contrast T1 FS image (F, thick arrows).

An MRI of the pelvis was performed for further evaluation and staging of the cervical cancer. MRI showed an endocervical infiltrative, altered signal intensity mass lesion involving the cervix, with a maximum length of 5.2 cm and cross-sectional dimensions of 4.9 × 3.5 cm (TR ×AP). The lesion showed a mildly hyperintense signal on T2-weighted and STIR images with marked diffusion restriction and heterogeneous post-contrast enhancement (Figure 1A-1F). The lesion showed parametrial extension without any involvement of the pelvic wall. The lesion was obliterating the posterior and potentially left lateral fornices inferiorly and was projecting into the upper vagina with no evident vaginal wall invasion. Superiorly, the lesion extended up to the lower uterine myometrium, especially along the left side. Anteriorly, there was no obvious loss of fat planes in the posterior serosa of the urinary bladder. Posteriorly maintained fat planes were observed with the rectum. There was significant cervical stenosis with resultant hydrometra (approximate collection of 160 cc) and an enlarged uterus (Figure 1B, 1C). Irregular thickening and nodular deposits were noted along the endometrial lining in contiguity with the cervical lesion, showing signal intensity similar to the lesion with restricted diffusion and heterogeneous enhancement (Figure 2A-2F). The differential diagnosis of primary malignant cervical neoplasm (FIGO stage IIB) with the superficial extension of the primary neoplasm along the endometrium and synchronous primary malignant neoplasms of the cervix and endometrium was considered.

**Figure 3:**
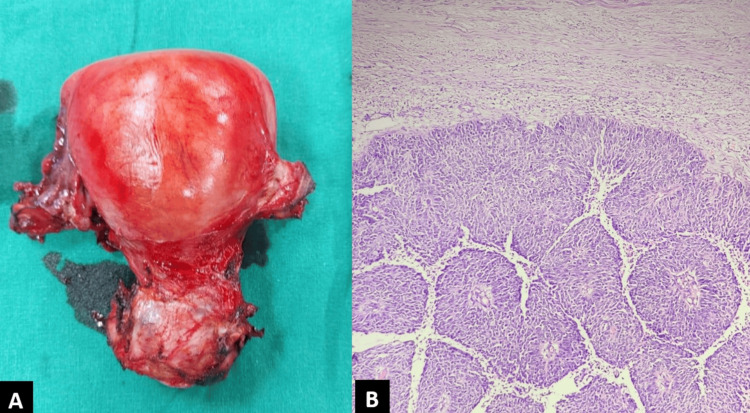
A, B: Post-radial hysterectomy gross specimen of the uterus (A) shows a bulky uterus with irregular cervical growth. Histopathological examination (B, H&E stain) showed tumor cells with marked nuclear atypia, inconspicuous nucleoli, and a moderate amount of cytoplasm. The diagnosis of poorly differentiated SCC (not otherwise specified) grade 3 was made. SCC, squamous cell carcinoma

The patient underwent a radical hysterectomy, and the histopathological examination confirmed the diagnosis of poorly differentiated nonkeratinizing squamous cell cervical carcinoma (histological grade 3) with contiguous extension of squamous cells to the lining of the uterine endometrium (Figure 3A, 3B). The lesion showed lymphovascular invasion and stromal invasion of more than 5 mm. Two right pelvic lymph nodes showed metastatic deposits. The HPV and p16 were found to be negative. The final diagnosis was poorly differentiated cervical SCC, FIGO stage III C1 (2018), with superficial spread of the SCC to the endometrium.

## Discussion

Cervical SCC, accounting for 70-80% of all cervical malignancies, is the most prevalent type of malignant tumor of the cervix [1]. Cervical cancer typically spreads directly into the uterine wall, either with or without parametrial involvement. It is extremely rare for SCC to grow superficially upward, replacing the epithelium on the surface without penetrating the myometrium or endometrial stroma beneath [1-3]. This type of cervical SCC is unique in that it can travel to the inner surface of the uterus and replace the endometrium with cancerous cells [1].

In the cases of superficially spreading cervical SCC that have been documented thus far, the endometrium has been the primary, often isolated, region affected [1-4]. The disease is usually limited to the endometrium without evidence of invasion, while invasive endometrial spread is less common, reported in approximately 18.5% of cases by Martín-Vallejo et al. in their literature review of 54 patients [2]. Nevertheless, there are not many examples in the literature that demonstrate endometrial extension along with unilateral or bilateral involvement of the ovaries, fallopian tubes, or both [2,4-7]. Additionally, Nakajima et al. described a case with superficial SCC spread to the ovaries, fallopian tube, endometrium, and also the greater omentum [4].

Fluhmann originally proposed diagnostic criteria in 1928 for the differentiation of primary and secondary endometrial squamous neoplasia, including in situ lesions [8]. This criterion is applied to verify the presence of superficial spread and rule out contemporaneous endometrial cancer. According to these guidelines, the diagnosis of primary endometrial SCC necessitates the absence of (a) coexisting endometrial adenocarcinoma; (b) a demonstrable connection between the endometrial tumor and the stratified squamous epithelium of the cervix; and (c) primary cervical SCC. This can be achieved through comprehensive histopathological examination and imaging studies, such as MRI or PET-CT. In the present case, the contiguity of the endometrial involvement with the primary cervical carcinoma was seen on MRI, and histopathological examination confirmed the diagnosis of SCC.

Martín-Vallejo et al. did a review of 54 cases of superficially spreading cervical carcinoma reported from 1956 to 2022, with age at presentation ranging from 44 to 78 years [2]. The most common presenting complaint was found to be post-menopausal vaginal bleeding and discharge. The 55-year-old female in the index case presented with a white vaginal discharge without bleeding, similar to that reported by Kushima et al. [5]. Yang et al. [9] and Anthuenis et al. [10] also reported hydrometra as a presenting complaint in their case reports, which was also found to be present in the MRI in our case. A previous history of radiation therapy followed by pelvic cobalt therapy and cervical stenosis with pyometra has been reported to favor the superficial spread of cervical SCC [2,11-13]. Endometrial extension rather than lateral spread of cervical cancer is reported to be associated with cervical stenosis [11,12], which was also present in our case. However, Bagde et al. found cervical stenosis in only five out of 25 cases in their literature review of superficially spreading cervical cancer and concluded that cervical stenosis may be an important factor, but not the only cause of this type of cervical cancer [13].

Chao et al. found HPV-p16 positivity in all endometrial and fallopian tube histopathological specimens of their patient, pointing toward the key role of HPV infection in the development of the superficial spread of SCC [11]. HPV as a causative factor for the superficial spreading of SCC has also been widely reported by other authors [2,5,14]. Du and Liao., in their case of cervical SCC with contiguous spread to the endometrium, found immunohistochemical expressions of p16, p63, and CK5/6 in the cervix and endometrium lesions, which suggests an etiological relationship between the two lesions and supports the idea that endometrial SCC is due to the spread of in situ cervical SCC [14]. Nicolás et al.’s multivariate analysis revealed that only a tiny percentage of cervical malignancies arise from a mechanism unrelated to HPV [15]. These HPV-negative tumors are more likely to metastasize to lymph nodes, are diagnosed at advanced stages, and have a dismal prognosis. A trend of HPV-p16-negative cases showing increased mortality and worse overall survival was also reported in other studies [16-18]. In the present case, HPV and p16 were found to be negative. However, the continuity of the cervical and endometrial lesions on imaging and histopathological examination strengthened the diagnosis of superficial spread of cervical SCC.

It is unknown if the stage, course of treatment, and prognosis are altered by this type of superficial spread. Martín-Vallejo et al. did a review of 54 patients, out of which 33 were lost for follow-up, two patients died of disease, and 19 were disease-free after six to 132 months [2]. In the index case, the patient unfortunately died a few days after surgery due to other complications. The latest FIGO staging (2018) and TNM staging (American Joint Committee on Cancer tumor (Version 9) [19]) do not encompass the superficial spread of cervical carcinoma to the uterine endometrium, fallopian tubes, and ovaries [20]. Consequently, prognostic importance and uniform management guidelines are lacking due to the unique nature of this pattern and limited literature data [14]. However, there have been many reports in the literature that have found the superficial endometrial spread of cervical SCC to be associated with a worse prognosis than primary endometrial SCC [2,14,20]. Future research efforts are needed to focus on further elucidating the molecular mechanisms underlying its distinct behavior and exploring targeted therapeutic approaches for a better prognosis.

## Conclusions

A superficially spreading SCC represents a unique entity within the spectrum of cervical SCCs, necessitating careful evaluation of preoperative imaging for proper patient management. Establishing the continuity of the lesion on imaging and histopathological testing is critical to confirm the presence of a superficial spread of cervical cancer. MRI evaluation, being nonionizing and noninvasive, offers a means to identify this rare form of cervical SCC spread. The occurrence of a superficial spread of nonkeratinizing SCC from the cervix to the endometrium is typically associated with a positive result for HPV in immunohistochemistry. However, in our instance, the result was negative for HPV, making it an unusual presentation for a case report.
